# Acetone Detection and Classification as Biomarker of Diabetes Mellitus Using a Quartz Crystal Microbalance Gas Sensor Array

**DOI:** 10.3390/s23249823

**Published:** 2023-12-14

**Authors:** Marcos Rodríguez-Torres, Víctor Altuzar, Claudia Mendoza-Barrera, Georgina Beltrán-Pérez, Juan Castillo-Mixcóatl, Severino Muñoz-Aguirre

**Affiliations:** Facultad de Ciencias Físico-Matemáticas, Benemérita Universidad Autónoma de Puebla, Avenida San Claudio y 18 Sur, Colonia San Manuel, Edificio FM1-101B, Ciudad Universitaria, Puebla 72570, Mexico; marcos.rodriguezt@alumno.buap.mx (M.R.-T.); valtuzar@fcfm.buap.mx (V.A.); cmendoza@fcfm.buap.mx (C.M.-B.); gbeltran@fcfm.buap.mx (G.B.-P.); jcastill@fcfm.buap.mx (J.C.-M.)

**Keywords:** quartz crystal microbalance, ultrasonic atomization, acetone biomarker, principal component analysis, discriminant analysis, diabetes mellitus

## Abstract

A gas sensor array was developed and evaluated using four high-frequency quartz crystal microbalance devices (with a 30 MHz resonant frequency in fundamental mode). The QCM devices were coated with ethyl cellulose (EC), polymethylmethacrylate (PMMA), Apiezon L (ApL), and Apiezon T (ApT) sensing films, and deposited by the ultrasonic atomization method. The objective of this research was to propose a non-invasive technique for acetone biomarker detection, which is associated with diabetes mellitus disease. The gas sensor array was exposed to methanol, ethanol, isopropanol, and acetone biomarkers in four different concentrations, corresponding to 1, 5, 10, and 15 µL, at temperature of 22 °C and relative humidity of 20%. These samples were used because human breath contains them and they are used for disease detection. Moreover, the gas sensor responses were analyzed using principal component analysis and discriminant analysis, achieving the classification of the acetone biomarker with a 100% membership percentage when its concentration varies from 327 to 4908 ppm, and its identification from methanol, ethanol, and isopropanol.

## 1. Introduction

Diabetes mellitus disease is a chronic health condition that affects a portion of the global population, including both young and adult individuals, with 6% of the adult global population developing this disease [1,2,3]. Diabetes mellitus is characterized by the dysregulation of various metabolic processes, mainly inadequate control of glucose blood levels. This illness is caused by low insulin production in blood, insulin resistance within the human body, overweight, obesity, or a combination of these factors [1]. Currently, diabetes mellitus is detected by the glucose level in blood tests, which are considered an invasive procedure. This procedure involves a finger prick or a blood draw from a vein, inducing physical discomfort, and on occasion, skin and vein injury in individuals undergoing diagnosis. Therefore, non-invasive methods for diabetes mellitus detection have been developed, wherein volatile organic compounds (VOCs)’ concentration levels are measured and analyzed from exhaled human breath, which is closely associated with the metabolic process underlying diabetes mellitus [2,4,5,6]. These VOCs are called biomarkers, and they are biological molecules present in blood, liquids or biological tissue, wherein their presence is a sign of normal or abnormal metabolic processes. Exhaled breath serves as a convenient and accessible medium for biomarker measurement. On the other hand, gas sensor arrays have emerged as a promising technology for the non-invasive detection of diabetes mellitus biomarkers, such as acetone (Ace). Some authors have reported that the concentrations are 2.2–22 ppm for type 1 diabetes patients and 1.76–9 ppm for type 2 diabetes patients [7]. Sensor arrays exhibit a high sensitivity to VOCs, including acetone, as well as other VOCs present in the human breath such as methanol (MeOH), ethanol (EtOH), and isopropanol (iPrOH); gas sensor arrays can quantify biomarkers’ concentration levels, and discriminate between them [8,9,10,11,12]. In the literature, there are reports of various types of devices that have been used for constructing gas sensor arrays, primarily for the detection of VOCs. Metal-oxide semiconductor (MOS), optical, surface acoustic wave (SAW), and quartz crystal microbalance (QCM) devices are commonly employed, since they can be combined with different sensing film materials. The sensing film is the compound that interacts with VOCs through a physical–chemical mechanism, while the device converts such an interaction into an electrical signal (voltage or current) that can be quantified [13,14,15,16].

In the present work, QCM devices were employed due to their advantages in accessibility, cost effectiveness, and suitability for gas detection via deposition of a specialized sensing film on the surface [17,18,19]. QCM operates as a gravimetric device, where a mass change on the QCM electrode surface is measured through shift frequency (the mass loading effect). The QCM sensitivity is determined using the Sauerbrey equation [20], where the resonant frequency is used as the main parameter. For QCM devices with resonant frequencies ranging from 5 to 20 MHz, their sensibilities vary from 17.7 to 1.1 $\mathrm{ng}\cdot g\cdot {\mathrm{cm}}^{-1}$, while for a 30 MHz resonant frequency, a value of 0.5 $\mathrm{ng}\cdot g\cdot {\mathrm{cm}}^{-1}$ is obtained. This wide range of sensitivity enables the measurement of mass changes from micrograms to nanograms. Furthermore, the combination of QCM and sensing films has shown potential for classifying VOCs [21,22,23].

Polymeric materials are commonly used as sensing films in gas sensor arrays for biomarkers’ detection [23,24]. In gas sensor arrays, sensing films with different chemical and physical characteristics are employed; this offers enhanced selectivity and classification of VOCs associated with several diseases [25,26,27,28,29,30,31,32,33,34,35]. In this study, we introduce a gas sensor array based on high-frequency QCM, as a non-invasive method for the detection and classification of biomarkers associated with diabetes mellitus disease. The gas sensor array was made using ethyl cellulose (EC), polymethylmethacrylate (PMMA), Apiezon L (ApL), and Apiezon T (ApT) as sensing films. These sensing films were selected because of their chemical characteristics such as the solubility parameter $\left(\delta \right)$ that measures the dispersion forces, dipole–dipole interactions, and hydrogen bonding, and is related to the affinity between the sensing film and the VOCs [36]. Sensing film thicknesses were obtained with an approximate value of 200 nm by the ultrasonic atomization method [37]. The gas sensor array was exposed to MeOH, EtOH, iPrOH, and Ace biomarkers, categorized as alcohol and ketone groups. The measurements were performed at a temperature $\left(T\right)$ of 22 °C and a relative humidity $\left(RH\right)$ of 20%. We studied the gas sensor array’s behavior toward the biomarkers used. Principal component analysis (PCA) and discriminant analysis (DA) were applied to the gas sensor responses, successfully classifying the Ace biomarker with a 100% membership percentage, while MeOH, EtOH, and iPrOH were classified with membership percentages of 45, 55, and 65%, respectively.

## 2. Theory

### Sensing Film Thickness Estimation Using the Sauerbrey Equation

The Sauerbrey equation is shown in Equation (1); it relates the frequency shift $\left(\Delta f\right)$ of the QCM resonator to the mass changes $\left(\Delta m\right)$ on its surface:

(1) $$\Delta f=-\frac{2f_{0}^{2}}{\sqrt{\rho_{q}\mu_{q}}}\frac{\Delta m}{A},$$

where $f_{0}$ is the resonant frequency, A is QCM electrode area, $\mu_{q}$ is the quartz shear module, and $\rho_{q}$ is the quartz density [20].

When the sensing film is deposited on the QCM electrodes, its thickness $\left(\Delta d\right)$ is related to its volumetric density $\left(\varrho_{P}\right)$, as shown in Equation (2).

(2) $$\varrho_{P}=\frac{\Delta m}{V}=\frac{\Delta m}{A\cdot \Delta d},$$

where V=A·Δd is the sensing film volume.

The sensing film thickness $\left(\Delta d\right)$ can be estimated by substituting Δm from Equation (1) into Equation (2); we therefore obtain Equation (3).

(3) $$\Delta d=-\frac{\sqrt{\rho_{q}\mu_{q}}}{2}\frac{\Delta f}{f_{0}^{2}\varrho_{P}}.$$

The ultrasonic atomization method [37] was used to deposit the sensing film over the QCM electrode. This method is based on the capillary wave theory, where a mechanical oscillation is generated by a piezoelectric device, thereby propagating waves on a liquid medium. These waves induce the breakup of capillaries, resulting in the formation of a very small droplet mist. The capillary wavelength $\left(\lambda \right)$ is inversely related to the oscillation frequency of the piezoelectric device, and λ is estimated using the Kelvin equation [38], which is shown in Equation (4).

(4) $$\lambda ={\left(\frac{8\pi \sigma }{\rho_{l}F^{2}}\right)}^{1/3},$$

where λ is in ${\mathrm{cm}}^{-1}$, σ is the surface tension of the liquid medium in dinas/cm, $\rho_{l}$ is the liquid medium density in g/mL, and F is the ultrasonic frequency in Hz. The crests of capillary waves on the liquid surface produce droplets that are correlated with λ. Furthermore, the droplet average diameter $\left(D_{p}\right)$ was calculated using Equation (5), as result of Lang’s research [39].

(5) $$D_{p}=0.34\cdot \lambda =0.34\cdot {\left(\frac{8\pi \sigma }{\rho_{l}F^{2}}\right)}^{1/3},$$

Finally, the gas biomarker concentration was calculated using Equation (6):

(6) $$C=\left(\frac{22.4T_{a}\rho_{s}V_{l}}{273M_{w}V_{c}}\right)\cdot 10^{3},$$

where 22.4 L/mol represents the ideal gas molar volume at standard conditions of 1 atm and a temperature of 273.15 K. $T_{a}$ denotes the chamber temperature, $V_{l}$ is the volume of the biomarker in its liquid state, $V_{c}$ represents the total volume of the Teflon chamber, and $\;\rho_{s}$ and $M_{w}$ are the biomarker density and molecular weight, respectively.

## 3. Materials and Methods

### 3.1. Materials

The materials used as sensing films were ethyl cellulose (EC, CAS 9004-57-3), polymethylmethacrylate (PMMA, CAS 9011-14-7), Apiezon L^®^ grease (ApL, CAS 1267-02-3), and Apiezon T^®^ grease (ApT, CAS 9064-45-3), all of them purchased from Sigma-Aldrich (St. Louis, MO, USA). The solvent chloroform was purchased from Supelco Analytical Products, J.T. Baker (Phillipsburg, NJ, USA), and Meyer Corporation (Vallejo, CA, USA). The biomarkers methanol (MeOH, CAS 67-56-1), ethanol (EtOH, CAS 64-17-5), isopropanol (iPrOH, CAS 67-63-0), and acetone (Ace, CAS 67-64-1) were purchased from Supelco Analytical Products, Sigma-Aldrich, Fermont (Monterrey, Mexico), and Sigma-Aldrich, respectively. All reagents were of analytical grade and used without any further purification.

### 3.2. QCM Sensors’ Construction

**Figure 1:**
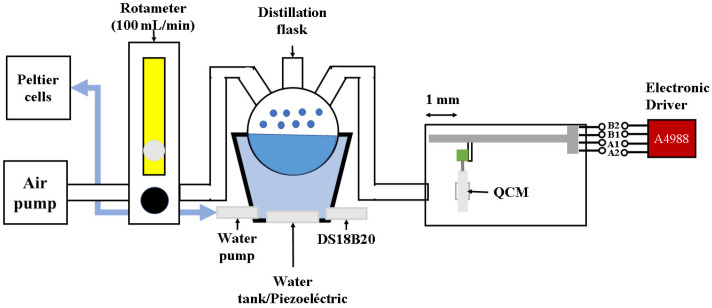
Ultrasonic atomization system used for the sensing film deposition.

The sensing films were deposited over the QCM electrodes (with a 30 MHz resonant frequency in fundamental mode with AT cut, and HC-49/U encapsulation) by an ultrasonic atomization system, as is shown in Figure 1. The system was composed of a 0.5 L water tank, and at its bottom a piezoelectric resonator was located, operating at a frequency of 1.3 MHz. The water temperature was kept at 22 °C with a water recirculatory system, which was connected to the water tank, and that used a water pump with a flow rate of 80 to 120 L/min. The recirculated water was directed to a chiller chamber formed of 2 Peltier cells (TEC-12706), and the water temperature was measured with a DS18B20 temperature sensor (Dallas Semiconductor, Dallas, TX, USA). The sensing film droplet mist was generated inside a distillation flask with 3 angled necks and a volume of 50 mL. The sensing film dissolutions were prepared with the following concentrations: EC at 10 mg/mL, PMMA at 2 mg/mL, ApL, and ApT at 1.5 mg/mL, all of which were diluted in chloroform. Two necks of the flask served as the air inlet and outlet, respectively, for the air–mist mixture. The droplet diameter was estimated using Equation (5), with chloroform values of $\rho_{l}=1.4832\;g/\mathrm{mL}$ and σ=27.14 dinas/cm, and a piezoelectric resonance frequency of F=1.3 MHz, resulting in $D_{p}=220\;\mu m$. An air pump was connected to a model P single flow tube rotameter (Aalborg, Orangeburg, NY, USA) with a scale from 100 to 1000 mL/min, which was set to deliver a constant air flow of 100 mL/min. The pump supplied air to the flask and the outlet was connected to a polyurethane pipe with a 4 mm inner diameter and a length of 20 cm, which transported the microdroplet mist to the QCM electrode. The QCM was mounted on a base moved by a linear stepper motor (with a steep angle of 18 °C) driven by an A4988 electronic driver, and the base and the linear stepper motor were placed inside the deposition chamber. The QCM electrode was aligned with the pipe outlet, maintaining a 1 mm separation distance. The whole system was managed with a PIC16F877A microcontroller and an interface developed on a personal computer.

Before the sensing film deposition, the QCM electrodes were cleaned by exposure to an ultraviolet–ozone chamber (Bioforce Nanoscience, Virginia Beach, VA, USA), and frequency scanning was employed to obtain the QCM resonant frequency. Finally, the Q factor was determined through analysis of the obtained resonance curve.

**Figure 2:**
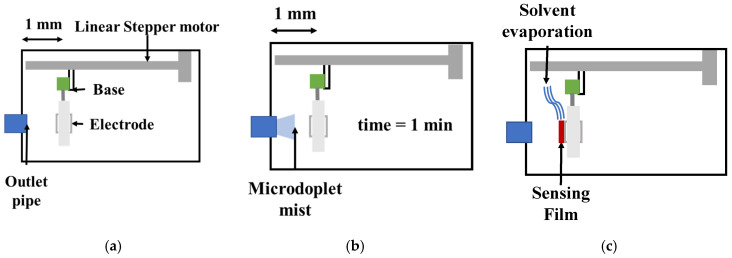
The sensing film deposition process of ultrasonic atomization: (**a**) the QCM is placed 1 mm away from the pipe outlet (blue); (**b**) the QCM (gray) is exposed to microdroplet mist for 1 min; and (**c**) the sensing film thickness (red) is obtained after the solvent’s evaporation.

The sensing film thickness was estimated by Equation (3) based on the QCM resonant frequency $f_{0}$ after the cleaning process. This allowed us to estimate the frequency shift of the deposited sensing film through the microdroplet mist. The sensing film deposition process is shown in Figure 2. In Figure 2a, it is shown that the QCM was mounted on a base with a linear stepper motor and displaced using an interface developed on a personal computer. In Figure 2b, we show that the QCM electrode was exposed to the microdroplet mist carried by an air flow of 100 mL/min for 1 min, and after that, the solvent was allowed to evaporate for another minute. Next, the scanning frequency was obtained, and Δf was the obtained as the result of mass change on the QCM electrode, enabling us to calculate the sensing film thickness, as shown in Figure 2c. This process was repeated the necessary number of times until the desired thickness was reached. Finally, the QCM was dismounted and rotated on the base to apply the deposition process to the opposite electrode.

### 3.3. Gas Sensor Response Measurement

**Figure 3:**
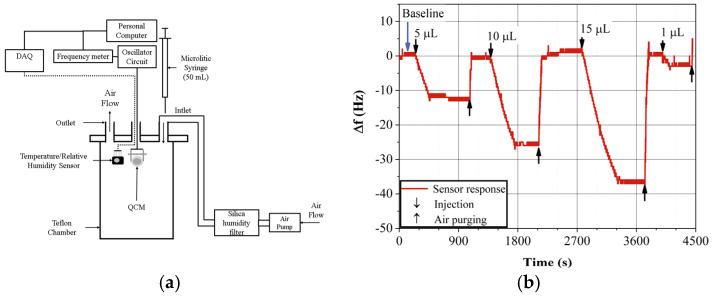
(**a**) Static measurement gas sensor response system and (**b**) ApL sensor exposed to iPrOH.

Figure 3a shows the setup of the static system used to measure the gas sensors’ responses. The system comprises a Teflon chamber (1 L volume). The gas sensors and a temperature-relative humidity digital sensor (HIH 8121 4 pins SIP, Honeywell, Charlotte, NC, USA) were placed inside the chamber. The QCM gas sensors were connected to an oscillator circuit and the frequency data were transmitted to a homemade frequency meter with 1 Hz resolution and sample rate of 1 datum per second [40]. The Δf data as well as the temperature and relative humidity were stored in and displayed on a personal computer [41].

The QCM gas sensor was placed inside the Teflon chamber, and it was closed. An inlet was used to introduce air into the chamber using a pump (3.2 rpm, HiLetGo, Shenzhen, China). The pumped air was reduced in relative humidity until 20% using a silica humidity filter, as is shown in Figure 3a; the temperature was kept at 22 °C. In Figure 3b, a typical gas sensor response curve is shown. At the beginning, when the internal conditions reached the equilibrium state, the gas sensor frequency remained constant; this frequency, called the baseline, was monitored for 5 min. After that, the liquid sample of the biomarker was injected into the chamber using a microliter syringe (50 µL, Hamilton, Reno, NV, USA). The liquid was left to evaporate and disperse within the chamber, the biomarker concentration was calculated using Equation (6), measured in parts per million (ppm). The sample injection process caused a frequency shift from the baseline when a second state of equilibrium was reached. The frequency of the gas sensor remained constant for another 5-min interval. Finally, the purge process was initiated to replace the gas–air mixture with air reduced in relative humidity. After this process, the gas sensor was cleaned, and its frequency returned to the baseline.

The gas sensors were exposed to four volume values (1, 5, 10, and 15 µL) of each biomarker, as shown in Table 1. For each concentration, the gas sensor response was measured five times under controlled conditions of 22±0.5 °C temperature and 20±2% relative humidity. Furthermore, the effect of the sensing films’ thickness was eliminated by constructing all the sensors with thicknesses close to 200 nm. The characterization process performed in the present work only corresponds to the calibration of the gas sensor array, as is described in other works in the literature [35,42,43].

## 4. Results and Discussion

### 4.1. Impedance Curves

**Figure 4:**
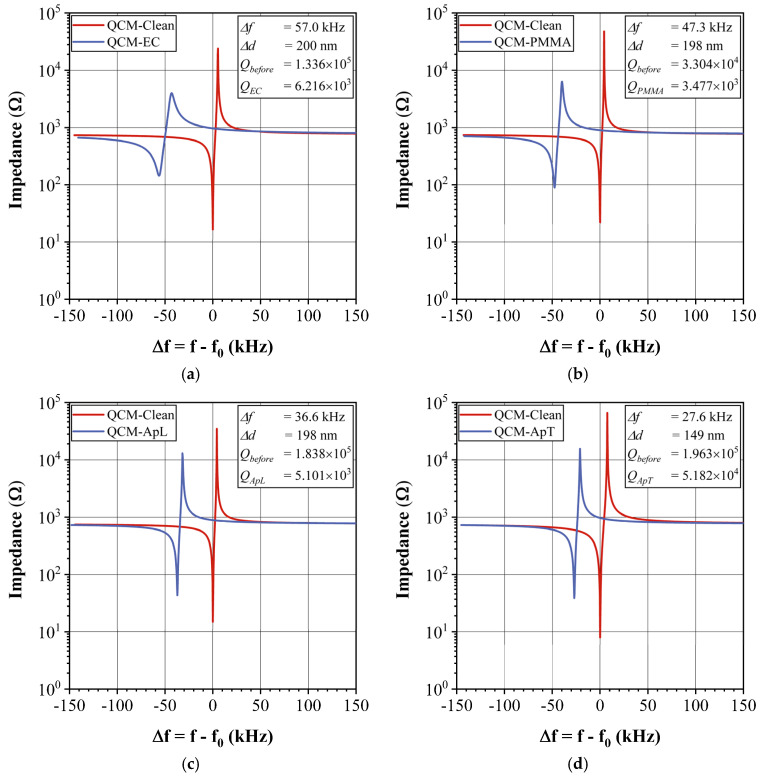
Impedance curves for the QCM deposited with the following sensing films: (**a**) EC, (**b**) PMMA, (**c**) ApL, and (**d**) ApT.

The sensing films (EC, PMMA, ApL, and ApT) deposited on the QCM electrodes by the ultrasonic atomization method produced a frequency shift. In Figure 4, all the clean crystals are represented by a red line curve centered at zero, while the sensing film deposited on the QCM electrodes is indicated by a blue curved line. The deposition of the EC sensing film is shown in Figure 4a, resulting in a frequency shift of Δf=57 kHz, corresponding to an average estimated thickness of Δd=200 nm. As a result of the EC deposition, the impedance at the resonant frequency peak changed from 16 to 150 Ω. Additionally, the resonance peak bandwidth broadened, as quantified by the Q factor. The Q factor measures the rate of energy storage and energy loss per oscillation cycle of the QCM. This parameter helps to determine whether the deposited QCM with the sensing film can be used as gas a sensor and to perform its characterization. For the EC sensing film, a ΔQ=5% was obtained, which is an adequate value, since we have observed that devices with ΔQ≈ 50% can still be used as gas sensors. In Figure 4b, a frequency shift of Δf=47.3 kHz is observed for the QCM device deposited with PMMA, with an impedance change at the resonant peak from 12 to 91 Ω, which means a ΔQ=10% (again, a suitable value). The PMMA sensing films’ resonant peaks maintained their shape and depth. In Figure 4c,d, the QCM devices with ApL and ApT grease sensing films are shown, respectively. The shapes of their resonance peaks remained unchanged, and they showed values of Δf=36.6 kHz and Δf=27.6 kHz, respectively, corresponding to ΔQ values of 0.36% and 26% for the sensors used. Even though ApT showed the highest ΔQ, its value was still adequate, as mentioned above.

Table 2 provides a summary of the frequency shift Δf and the thickness changes Δd for each of the electrodes located at both sides of the QCM $\left(E_{1},E_{2}\right)$ produced by the sensing films’ deposition. Different concentration values were used during the deposition process, resulting in different rates of thickness increase in the sensing films over time. For instance, the ApL sensing film exhibited an increase rate of 2 nm/min, while the ApL sensing film increased at a rate of 25 nm/min. Moreover, it is worth mentioning that the thickness increment of all the sensing films remained relatively constant. When the first electrode was deposited, the thickness values ranged from 99 to 133 nm. However, when the second electrode was deposited, the values varied from 36 to 133 nm for all the sensing films. This deposition process allowed us to achieve the desired total thickness. The total thicknesses of the sensing films deposited for the four compounds were quite similar, showing the suitability of the ultrasonic atomization method.

### 4.2. QCM Sensing Film Surface Roughness Analysis

**Figure 5:**
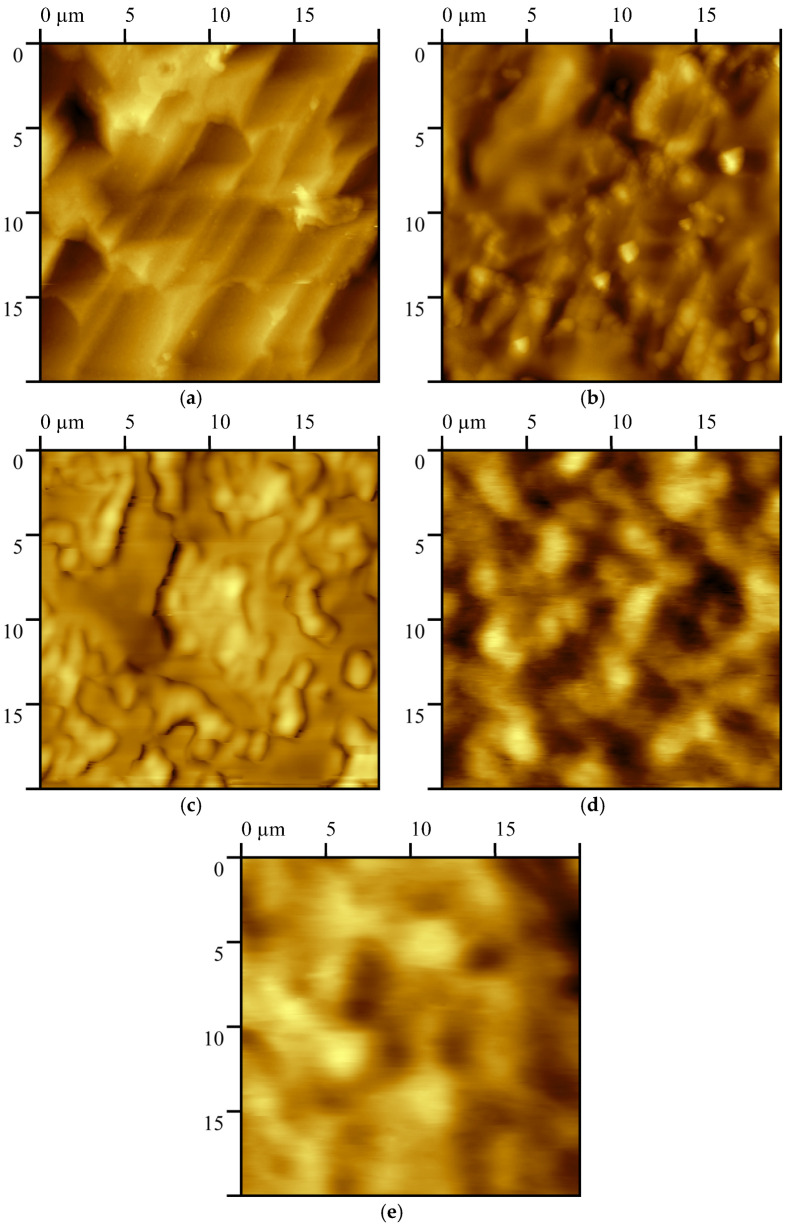
AFM micrography surface of (**a**) QCM without a sensing film (a naked electrode), and QCM covered with sensing films of (**b**) EC, (**c**), PMMA, (**d**) ApL, and (**e**) ApT.

Four different compounds were used as sensing films, deposited by ultrasonic atomization. The microdroplet mist was directed towards the QCM electrodes, as was explained in the Section 3.2. As a result, the morphologies of the sensing films over the QCM electrodes has specific characteristics that can lead to the increased sensitivity of the gas sensors compared with other deposition methods [37]. The ultrasonic atomization reduces the attraction forces between the polymer chains [44]. Therefore, the polymer dissolved in the microdroplets that are deposited over the QCM surface produced a porous sensing film for each deposited layer. A comparison of micrographies was performed for the QCM electrodes with and without sensing films. The topographies are shown in Figure 5. The micrographies were obtained from a 20×20 μm area using an atomic force microscope (AFM Park XE7, Park Systems Corp., Suwon, Republic of Korea) in non-contact mode. The naked electrode is shown in Figure 5a, where it can be observed that its surface is not completely flat, and exhibited a surface mean roughness of $S_{a}=123\;\mathrm{nm}$. Figure 5b shows the distribution of the EC sensing film over the QCM electrode, where there are high (bright) and low (dark) areas, indicating variations in film porosity due to the deposition method. In ultrasonic atomization, we deposited layer by layer, while in drop casting, the film was deposited all at once [25]. The surface mean roughness was estimated as $S_{a}=76\;\mathrm{nm}$. In Figure 5c, the distribution of the PMMA sensing film is shown. Compared with the EC sensing film, PMMA presented agglomeration in specific zones. The film porosity increases the effective area, allowing the sorption of biomarker sample molecules. The PMMA surface mean roughness was $S_{a}=46\;\mathrm{nm}$, which is quite a low value compared with the naked electrode. The distributions of the ApL and ApT sensing films are shown in Figure 5d,e. The two sensing films showed similar characteristics, resulting in similar shapes over the QCM surface. Both sensing films exhibited a kind of semi-spherical shape with high zones; this increases the effective surface area. The surface mean roughness was estimated as $S_{a}=77\;\mathrm{nm}$ for the ApL sensing film and $S_{a}=56\;\mathrm{nm}$ for the ApT one. In summary, the sensing films showed different shapes over the QCM electrode: the EC and PMMA sensing films have a porosity area, whereas the ApL and ApT sensing films covered the entire electrode, without porosity zones [25].

### 4.3. Sensor Array Characterization

**Figure 6:**
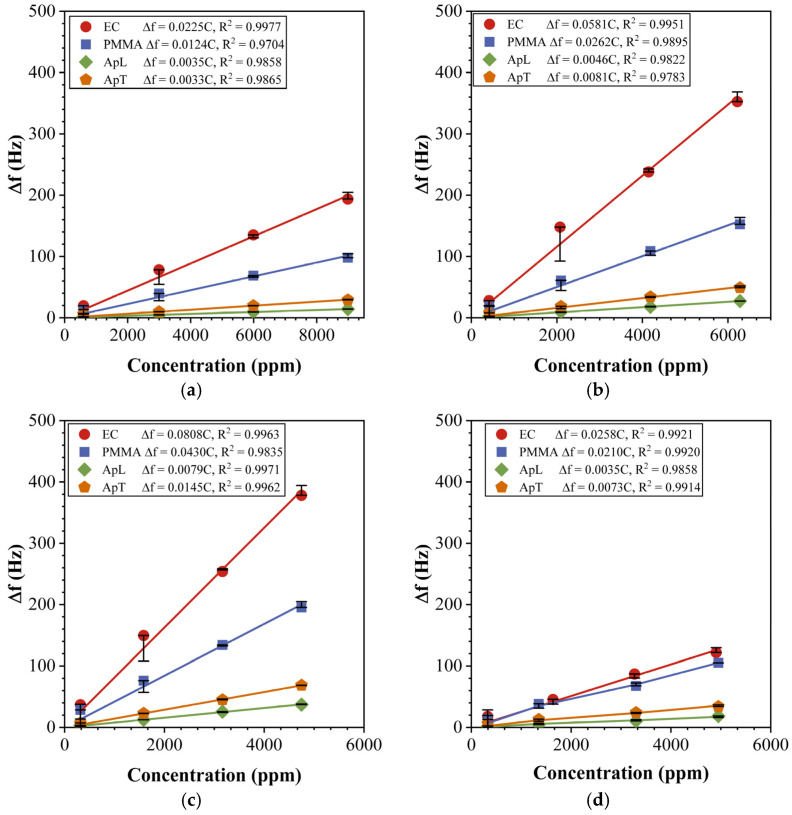
Gas sensor responses for QCM with EC, PMMA, ApL, and ApT sensing films; these were exposed to (**a**) MeOH, (**b**) EtOH, (**c**) iPrOH, and (**d**) Ace, under conditions of T = 20 °C and HR = 20%.

In Section 3.3, we described the exposure of QCM gas sensors to MeOH, EtOH, iPrOH, and Ace gas biomarkers under constant conditions of T = 22 °C and RH = 20%. The gas sensors were measured five times for each concentration. In Figure 6, we show the behavior of the sensor responses in the function of the measured samples’ concentrations. In general, a linear relationship can be observed for all the sensors. In Figure 6a, we show a gas sensor array exposed to MeOH. The EC sensor presented the highest response of Δf=194 Hz, while the PMMA sensor showed a response of Δf=98 Hz, which is the sensor with the second-highest response within the gas sensor array. The sensor with the third-highest response was the ApT sensor with Δf=28 Hz, and the sensor with the lowest response was the ApL one, with Δf=19 Hz. All the above gas sensor responses were obtained at a MeOH concentration of 8966 ppm, which was the maximum measured concentration for this sample. The response to the EtOH biomarker is shown in Figure 6b, and we can observe that for all the gas sensors the response is higher than in the previous case. The gas sensor response pattern remained the same as when exposed to the MeOH biomarker. The highest response was observed on the QCM with an EC sensing film, followed by PMMA, ApT, and ApL in decreasing order. As mentioned above, a linear relationship was observed between Δf and C for all the concentration ranges (see Table 1). For this reason, the data were fitted to a linear function, where the slope is related to the gas sensor sensitivity $\left(S\right)$ in Hz/ppm. The fitted functions of the gas sensors for each compound are shown in the label within Figure 6. In general, the obtained responses for the Ace are the lowest, as is shown in Figure 6d.

The sensor with the highest sensitivity to EtOH was the EC one, with $S_{EC}=0.0581\;\mathrm{Hz}/\mathrm{ppm}$. The second sensor with more affinity to EtOH was PMMA, with $S_{PMMA}=0.0262\;\mathrm{Hz}/\mathrm{ppm}$, followed by ApT, with $S_{ApT}=0.0081\frac{\mathrm{Hz}}{\mathrm{ppm}},$ and finally ApL, with $S_{ApL}=0.0046\;\mathrm{Hz}/\mathrm{ppm}$. In general, EC is the sensor with the highest sensitivity; therefore, using its response, taking into account the resolution of the frequency meter (1 Hz) and the measurement noise, we calculated the limit of detection (LOD) to be 133 for MeOH, 52 for EtOH, 37 ppm for iPrOH, and 116 ppm for Ace. We obtained the different sensitivities of the gas sensor array used in this work, which enabled a unique fingerprint for each of the four biomarkers. When our sensors are compared with other ones in which an organic sensing film was employed, we found very good performance using our devices. For instance a chitosan sensing film achieved $S_{Ace}=0.0240\;\mathrm{Hz}/\mathrm{ppm}$ and $S_{iPrOH}=0.0493\;\mathrm{Hz}/\mathrm{ppm}$, a metal organic framework achieved $S_{Ace}=0.0008\;\mathrm{Hz}/\mathrm{ppm}$, and nanostructure-modified materials achieved $S_{Ace}=0.00003\;\mathrm{Hz}/\mathrm{ppm}$, which are smaller than the values achieved by our devices [45,46,47]. Even though our sensitivities are high, the results suggest that it is necessary to increase the sensitivity of gas sensor arrays in order to be able to quantify lower concentrations, especially for Ace.

**Figure 7:**
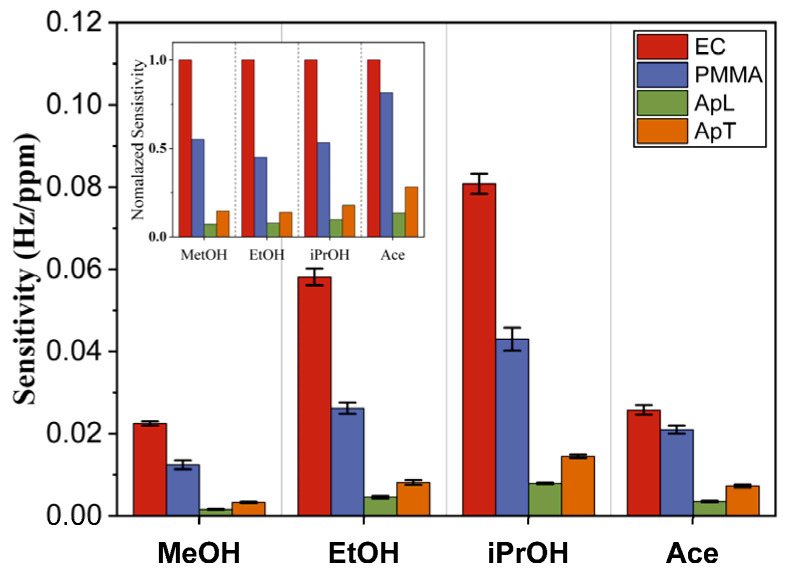
Gas sensor array response patterns for EC, PMMA, ApL, and ApT when they are exposed to MeOH, EtOH, iPrOH, and Ace.

The sensitivity patterns are shown in Figure 7. The EC sensor showed the highest sensitivity, followed by the QCM with a PMMA sensing film. The QCM with ApL and ApT sensing films exhibited the lowest affinity for the four biomarkers. When we compared their sensitivities to Ace, we observed that they are located between those of MeOH and EtOH, remaining lower than the sensitivities of iPrOH. It is worth mentioning that a gas sensor’s sensitivity is related to the affinity between the sensing film and the biomarker. One way to measure this affinity is through the solubility parameter, where a higher affinity exists between the sensing film and the biomarker when their parameters’ difference is small. The solubility parameter of each sensing film in the gas sensor array is as follows: $\delta_{EC}=18.82{\;\mathrm{MPa}}^{1/2}$, $\delta_{PMMA}=20.87{\;\mathrm{MPa}}^{1/2}$, and $\delta_{ApL}=15.95{\;\mathrm{MPa}}^{1/2}$. As for the biomarkers, their solubility parameters are $\delta_{MeOH}=29.61{\;\mathrm{MPa}}^{1/2}$, $\delta_{EtOH}=26.52{\;\mathrm{MPa}}^{1/2}$, $\delta_{iPrOH}=23.58{\;\mathrm{MPa}}^{1/2}$ and $\delta_{Ace}=19.94{\;\mathrm{MPa}}^{1/2}$. The δ values support the results indicating that the EC sensor has the highest sensitivity, followed by the PMMA sensor. The ApL and ApT sensors showed similar sensitivities; therefore, we can assume that their properties should be similar in terms of affinity (Figure 7) [36,48]. The EC sensing film contains ethoxy groups from 47% to 48%. The ethylation degree is associated with the solubility to solvents; therefore, the EC sensing film presents high affinity to the four biomarkers [49]. The PMMA sensing film interacts with the alcohols mainly through the hydrogen bond, due to the hydroxyl groups $\left(-OH\right)$ and the hydrocarbon chain length. On the other hand, The PMMA presents polar interactions with the Ace biomarker, since it is a polar compound distinguished for being a non-hydrogen donor [50]. Finally, ApL and ApT are long hydrocarbon chains and have a high molecular weight. They are considered non-polar compounds that present low interaction with polar compounds such as alcohols and ketones [48]. However, an important aspect is the form of their response pattern. In the inset of Figure 7, it becomes evident that the alcohols’ pattern is quite similar, and the Ace pattern is different, which suggests that it is possible to distinguish Ace using pattern recognition techniques such as PCA and DA.

### 4.4. PCA and DA Application for Acetone Classification

**Figure 8:**
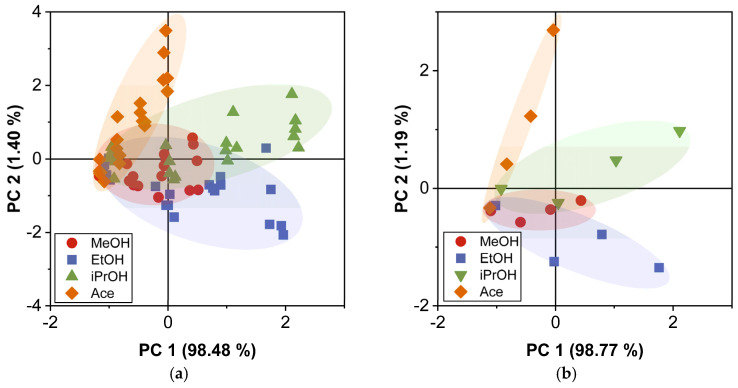
PCA results for (**a**) gas sensor raw responses and (**b**) gas sensor average responses at T=22 °C and RH=20%.

The objective in this paper was to classify and identify the acetone biomarker among MeOH, EtOH, and iPrOH biomarkers, since Ace is the key compound for diabetes mellitus detection. The gas sensor responses to these four biomarkers are shown in the results presented in Figure 7. The gas sensor array presented specific behavior in response to each biomarker, with similar responses observed in the lower concentration of each biomarker (see Table 1). Therefore, PCA was used to separate the data correlated within the gas sensor responses, and project them onto new axes where the gas sensor responses are uncorrelated. The gas sensor response matrix was constructed using the frequency shift of the four sensors $\left(\Delta f_{EC},\;\Delta f_{PMMA},\;\Delta f_{ApL},\;\;\Delta f_{ApT}\right)$. As a result of application of PCA, we obtained four principal components (PC). In Figure 8, PCA results are shown (only for PC1 and PC2) applied to the raw data and the average of all responses for each biomarker. We can distinguish the data groups for each biomarker: the red points refer to MeOH, the blue to EtOH, the green to iPrOH, and the orange to Ace. In both cases, the total variance found was more than 99%, considering PC1 and PC2. In general, in Figure 8a, we can observe that the alcohol groups are located in quadrants I, III and IV, while Ace group is located in quadrant II. Additionally, there is a clear separation of Ace from alcohol groups. However, for low concentrations, there is an overlap specially with the MeOH group that is located quite close to Ace, probably leading to confusion in their classification. Figure 8b shows a decrease in data dispersion due to the estimation of average responses for concentration groups and biomarkers. The overlapping of the groups also decreases, and the Ace separation becomes clearer.

**Figure 9:**
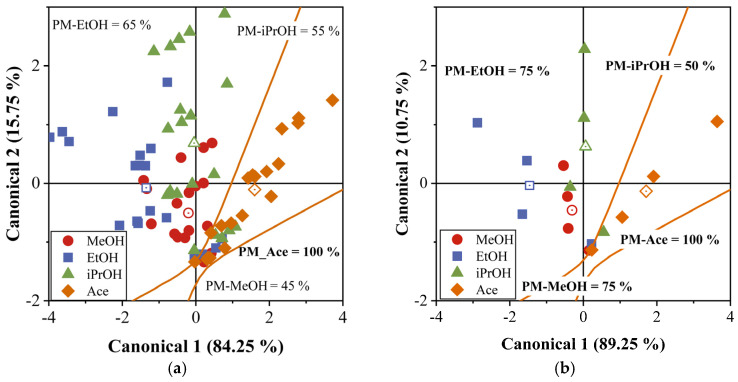
DA applied to PCA data: (**a**) gas sensor raw responses and (**b**) gas sensor average responses. The membership percentage is shown for MeOH, EtOH, iPrOH, and Ace biomarkers. Also, a quadratic classifier was fitted using the Ace and MeOH data.

To achieve better classification, we used a quadratic discriminant classifier (QDA) using the PCA results (Figure 9). The orange line was used as a boundary to distinguish MeOH, EtOH, and iPrOH from the Ace biomarker. This curved line was used as a quadratic classifier for the Ace group. The implicit function associated with this classifier was estimated using Equation (7).

(7) $$K+\left(\begin{array}{cc} C1 & C2 \end{array}\right)\cdot L+\left(\begin{array}{cc} C1 & C2 \end{array}\right)\cdot Q\cdot \left(\begin{array}{c} C1\\ C2 \end{array}\right)=0,$$

where K, L, and Q are constant, linear, and quadratic coefficients, respectively, which were calculated using the classifier in MatLab R2023a software (academic license 41049758). C1 and C2 are the canonical variable values for the gas sensor responses (raw data and average data). Even though we calculated all the combinations between the different pairs of groups, in Figure 9, only the fitted curve for the Ace and MeOH groups is shown, because these are the ones with the highest probability of being confused. In both cases, those of raw data and average data, the membership percentage (MP) of the Ace biomarker is 100%, which means that the QDA allowed for the clear and accurate classification of the Ace biomarker, which showed no confusion with MeOH, EtOH, and iPrOH alcohols. Conversely, the membership percentages for MeOH (MP-MeOH), EtOH (MP-EtOH), and iPrOH (MP-iPrOH) were 45%, 65%, and 55%, respectively, although for the case of average data, these values were higher, at 75%, 75%, and 50%, owing to the smaller dispersion (see Figure 9b). This indicates that the alcohol groups were relatively easily confused with each other. Finally, we can say that with the gas sensor array used, we successfully classified the Ace biomarker in a mixture of different biomarkers frequently found in exhaled breath.

## 5. Conclusions

The use of ultrasonic atomization as a sensing film deposition method enabled similar thicknesses of the EC, PMMA, ApL, and ApT sensing films to be obtained. Additionally, the topography of the deposited sensing films exhibited a uniform distribution over the QCM electrodes, with porosity zones.

The impedance curves revealed that the resonance peak maintained its shape after the deposition of sensing films, with a ΔQ of less than 25% observed between the naked QCM and the QCM with a sensing film. The thickness of all the deposited sensors was approximately 200 nm.

The gas sensor responses were analyzed using PCA and DA. As a result, the MP for Ace was 100%, while the other biomarkers had an MP between 45 and 65%. This suggests that such biomarkers can be confused with each other. Therefore, it is advisable to incorporate a fifth sensing film into the gas sensor array, one with high affinity to the Ace biomarker, for better recognition at even lower concentrations than the one achieved with the LOD reached in the present work, which was 116 ppm.

## Figures and Tables

**Table 1:** Summary of biomarkers’ volumes and their concentrations $\left(C\right)$, calculated under conditions of T = 22 °C and RH = 22%.

Volume	$C_{MeOH}$	$C_{EtOH}$	$C_{iPrOH}$	$C_{Ace}$
µL	(ppm)	(ppm)	(ppm)	(ppm)
1	598	414	317	327
5	2992	2072	1583	1636
10	5983	4144	3165	3272
15	8975	6216	4748	4908

**Table 2:** Summary of gas sensors deposited by ultrasonic atomization, where $E_{1}$ and $E_{2}$ are the electrodes located at both sides of the QCM, ρ is the density of the sensing film, $\Delta f_{1}$ and $\Delta f_{2}$ are the frequency shift for each electrode, $\Delta d_{1}$ and $\Delta d_{2}\;$ are the thickness of each electrode, $\Delta f_{T}$ is the total frequency shift (of both electrodes), and $\Delta d_{T}$ is the total thickness of the sensing film for both electrodes.

Sensing Film	ρ (g/cm^3^)	$E_{1}$		$E_{2}$		$\Delta f_{T}$ (kHz)	$\Delta d_{T}$ (nm)	Thickness Increase Rate Δd/Δt (nm/min)
		$\Delta f_{1}$ (kHz)	$\Delta d_{1}$ (nm)	$\Delta f_{2}$ (kHz)	$\Delta d_{2}$ (nm)			
EC	1.14	28.1	99	28.9	101	57.0	200	20
PMMA	1.18	23.5	99	23.8	100	47.3	198	2
ApL	0.896	19.1	105	17.5	96	36.6	201	5
ApT	0.912	21.0	113	6.6	36	27.6	149	25

## Data Availability

The data presented in this study are available on request from the corresponding author.
